# Book Reviews

**DOI:** 10.1308/003588412X13171221591178

**Published:** 2012-05

**Table 1a:** 

EXTENT P/H	608 pages, Hardback
PRICE/ISBN	£70.00 9780199543328
PUBLISHER	Oxford University Press (Oxford), 2010
REVIEWER	Nick Newton
STAR RATING	****

The latest in the Oxford Desk Reference series looks at major trauma and follows the traditional format for trauma texts, starting with epidemiology and pre-hospital before moving on to the primary survey topics and proceeding logically through investigations, secondary survey and then specific topics. The book is comprehensive with regard to the range of topics addressed. Chapters on bariatric trauma, chemical, biological, radiological and nuclear injuries, psychology and rehabilitation in trauma are useful additions to this textbook.

The book is well laid out with each topic appearing in a logical manner and all the information displayed in an easy- to-access manner. However, the many blank pages make the book feel a little disjointed. The subject matter is covered in such a way as to be accessible but the text provides sufficient detail for the expert reader. The contributing authors are all experts in their field, which comes across well in the text.

The diagrams and pictures complement the text well but are a bit small and at times confusing. The decision making algorithms that are included are excellent. This book will appeal most to a specialist in trauma management who needs information on areas outside his or her own field.

## Oxford Desk Reference: Major Trauma

JASON SMITH, IAN GREAVES, KEITH M PORTER

| | |
|---|---|
| **EXTENT P/H** | 608 pages, Hardback |
| **PRICE/ISBN** | £70.00 9780199543328 |
| **PUBLISHER** | Oxford University Press (Oxford), 2010 |
| **REVIEWER** | Nick Newton |
| **STAR RATING** | **** |

The latest in the Oxford Desk Reference series looks at major trauma and follows the traditional format for trauma texts, starting with epidemiology and pre-hospital before moving on to the primary survey topics and proceeding logically through investigations, secondary survey and then specific topics. The book is comprehensive with regard to the range of topics addressed. Chapters on bariatric trauma, chemical, biological, radiological and nuclear injuries, psychology and rehabilitation in trauma are useful additions to this textbook.

The book is well laid out with each topic appearing in a logical manner and all the information displayed in an easy-to-access manner. However, the many blank pages make the book feel a little disjointed. The subject matter is covered in such a way as to be accessible but the text provides sufficient detail for the expert reader. The contributing authors are all experts in their field, which comes across well in the text.

The diagrams and pictures complement the text well but are a bit small and at times confusing. The decision making algorithms that are included are excellent. This book will appeal most to a specialist in trauma management who needs information on areas outside his or her own field.

## How to Operate

MATTHEW STEPHENSON

| | |
|---|---|
| **EXTENT P/H** | 288 pages, Paperback and DVD |
| **PRICE/ISBN** | £74.99 9780470657447 |
| **PUBLISHER** | Wiley-Blackwell (Oxford), 2011 |
| **REVIEWER** | Katy Valentine |
| **STAR RATING** | ***** |

*How to Operate* is a well-presented DVD/book combination aimed at medical students to surgical trainees, with an emphasis on MRCS candidates. 40 common open surgical procedures are covered, with approximately 10 hours of video providing appropriately aimed narrative and useful tips from consultant surgeons. In addition, anatomical diagrams and radiographs are superimposed to highlight key points.

The book complements the DVD well, with a chapter corresponding to each video. Extra chapters on consent, patient safety, etc, are also very applicable to work and exams. At the same size as the DVD case, the book can easily be slipped into a work bag and used as a standalone resource.

At a time when service provision sometimes seems to take priority over training, this learning resource offers a valuable opportunity to gain familiarity with an operation by watching the video and reading the book chapter prior to making it to the theatre list, allowing the most to be made of limited time in theatre. *How to Operate* fills a niche in the market for core surgical trainees and I am sure our registrars and consultants will be wondering why there wasn't anything similar available earlier!

## History of British Intensive Care, c.1950–c.2000

LA REYNOLDS, EM TANSEY

| | |
|---|---|
| **EXTENT P/H** | 157 pages, Paperback |
| **PRICE/ISBN** | £6.00 9780902238756 |
| **PUBLISHER** | Queen Mary (London), 2011 |
| **REVIEWER** | Ron Jones |
| **STAR RATING** | **** |

The Wellcome Trust created a history of medicine group in 1990 and has organised regular 'Witness Seminars' on a variety of topics, the transcripts of which provide an oral history of 20th-century British biomedicine.

The latest seminar concerns the development of British intensive care, which had its roots in wartime experiences of triage, transfusion and team work and was bought into focus in the 1950s by the polio epidemic and then the development of cardiac surgery. For those with an interest in history or intensive care this is an enjoyable and easy read. Many of the pioneers, including Ron Bradley, Tony Gilbertson and Iain Ledingham, contributed to the seminar, which really brings it to life. Often it is the casual anecdote that draws things together. For instance, I did not know that among the factors coinciding in the 1951–1952 polio epidemic in Copenhagen, which led to significant advances in assisted ventilation, was the fact that the Danish brewers Carlsberg already had experience in pH analysis for beer production and that this was developed by Radiometer into expertise in blood gas analysis.

The fundamental question: 'does intensive care do any good or has it developed as a means of allowing surgeons

Book Reviews May 2012.indd 290 13/04/2012 11:38:35

and physicians to go to bed and give anaesthetists something to do?' is addressed with candour (read the book to find the answer; it's complicated). Accepting that intensive care is here to stay, and the specialty was granted faculty status by the GMC in 2010, this excellent review also covers the role of specialist nursing care, monitoring equipment, scoring systems and national audit. For its target audience it comes highly recommended.

## Resilience Engineering in Practice: A Guidebook

ERIK HOLLNAGEL, JEAN PARIÈS, DAVID D WOODS, JOHN WREATHALL

| | |
|---|---|
| **EXTENT P/H** | 322 pages, Hardback |
| **PRICE/ISBN** | £60.00 9781409410355 |
| **PUBLISHER** | Ashgate (Farnham), 2011 |
| **REVIEWER** | Denis Wilkins |
| **STAR RATING** | **** |

It has become increasingly apparent that as leaders of clinical teams and services, surgeons will be at a disadvantage unless they understand the concepts and principles enshrined within the term human factors. Such theory underpins a number of rather difficult and somewhat neglected areas of practice such as team working, professionalism and leadership. As clinicians take on more responsibility for shaping the systems within which they work, it becomes essential that they assimilate the experiences of other professionals working in not dissimilar, high-risk disciplines.

This book brings to the table a rich mix of relevant knowledge, anecdote, science and applied psychology. It comprises 18 chapters written by specialist psychologists, engineers, doctors and aviation experts, and links their expertise to the practical aspects of service delivery in a safe but efficient manner. There is a fair amount of science, which I quite liked and will be of practical use; for example, the data on fatigue and performance is clearly relevant to shift designs in clinical medicine. The concept of adaptive vs adapted systems provides much food for thought, particularly when it comes to training and simulation.

Some chapters are easier to read than others and I suspect that many of us will find some of the psychological and management constructs hard going. But overall this is a well-produced and edited book, which will prove a good investment of the time needed to digest it properly. It should be obligatory reading for all medical leaders and managers.

## Handbook of Surgical Consent

RAJESH NAIR, DAVID J HOLROYD

| | |
|---|---|
| **EXTENT P/H** | 768 pages, Paperback |
| **PRICE/ISBN** | £34.99 9780199595587 |
| **PUBLISHER** | Oxford University Press (Oxford), 2011 |
| **REVIEWER** | Sian Pugh |
| **STAR RATING** | *** |

Few doctors have survived their junior years without a copy of the much beloved Oxford Handbook of Clinical Medicine or 'cheese and onion' as it is colloquially known. This latest addition to the Oxford Medical Handbook series is aimed at surgical trainees to offer practical guidance on the principles of consent alongside procedure-specific information of the risks and benefits.

The book is divided into specialty-specific chapters that allow the reader to find their subject of interest readily. The concise manner in which it is written permits coverage of a broad range of procedures with well replicated diagrams adding clarity to the text. Complications are comprehensively listed. However, some readers may find the lack of guidance on incidence of these for the majority of procedures unhelpful.

This book certainly attempts to tackle a difficult and challenging topic. The concept that a book can outline what should be included on a surgical consent form for an individual patient may seem impossible to many surgeons. It must be remembered that consent is heavily dependent on the individual patient and so this book should be used as a basis rather than an exhaustive text. Provided it is used with this degree of caution it should be welcomed by trainees as an adjunct to surgical training.

Book Reviews May 2012.indd 291 13/04/2012 11:38:36
