# Supplementary material for: Book Reviews
Source: Ann R Coll Surg Engl. 2012 May;94(4):290–1. doi: 10.1308/003588412X13171221591178 (PMC3957523; doi:10.1308/003588412X13171221591178)
Supplement: Related texts

# Sub article: How to Operate

**Authors:** Matthew Stephenson

**Table 1b:** 

EXTENT P/H	288 pages, Paperback and DVD
PRICE/ISBN	£74.99 9780470657447
PUBLISHER	Wiley-Blackwell (Oxford), 2011
REVIEWER	Katy Valentine
STAR RATING	****

*How to Operate* is a well-presented DVD/book combination aimed at medical students to surgical trainees, with an emphasis on MRCS candidates. 40 common open surgical procedures are covered, with approximately 10 hours of video providing appropriately aimed narrative and useful tips from consultant surgeons. In addition, anatomical diagrams and radiographs are superimposed to highlight key points.

The book complements the DVD well, with a chapter corresponding to each video. Extra chapters on consent, patient safety, etc, are also very applicable to work and exams. At the same size as the DVD case, the book can easily be slipped into a work bag and used as a standalone resource.

At a time when service provision sometimes seems to take priority over training, this learning resource offers a valuable opportunity to gain familiarity with an operation by watching the video and reading the book chapter prior to making it to the theatre list, allowing the most to be made of limited time in theatre. *How to Operate* fills a niche in the market for core surgical trainees and I am sure our registrars and consultants will be wondering why there wasn’t anything similar available earlier!

# Sub article: History of British Intensive Care, C.1950-C.2000

**Authors:** LA Reynolds, EM Tansey

**Table 1:** 

EXTENT P/H	157 pages, Paperback
PRICE/ISBN	£6.00 9780902238756
PUBLISHER	Queen Mary (London), 2011
REVIEWER	Ron Jones
STAR RATING	****

The Wellcome Trust created a history of medicine group in 1990 and has organised regular ‘Witness Seminars’ on a variety of topics, the transcripts of which provide an oral history of 20th-century British biomedicine.

The latest seminar concerns the development of British intensive care, which had its roots in wartime experiences of triage, transfusion and team work and was bought into focus in the 1950s by the polio epidemic and then the development of cardiac surgery. For those with an interest in history or intensive care this is an enjoyable and easy read. Many of the pioneers, including Ron Bradley, Tony Gilbertson and Iain Ledingham, contributed to the seminar, which really brings it to life. Often it is the casual anecdote that draws things together. For instance, I did not know that among the factors coinciding in the 1951–1952 polio epidemic in Copenhagen, which led to significant advances in assisted ventilation, was the fact that the Danish brewers Carlsberg already had experience in pH analysis for beer production and that this was developed by Radiometer into expertise in blood gas analysis.

The fundamental question: ‘does intensive care do any good or has it developed as a means of allowing surgeons and physicians to go to bed and give anaesthetists something to do?’ is addressed with candour (read the book to find the answer; it’s complicated). Accepting that intensive care is here to stay, and the specialty was granted faculty status by the GMC in 2010, this excellent review also covers the role of specialist nursing care, monitoring equipment, scoring systems and national audit. For its target audience it comes highly recommended.

# Sub article: Resilience Engineering in Practice: A Guidebook

**Authors:** ERIK Hollnagel, Jean Pariés, David D Woods, John Wreathall

**Table:** 

EXTENT P/H	322 pages, Hardback
PRICE/ISBN	£60.00 9781409410355
PUBLISHER	Ashgate (Farnham), 2011
REVIEWER	Denis Wilkins
STAR RATING	****

It has become increasingly apparent that as leaders of clinical teams and services, surgeons will be at a disadvantage unless they understand the concepts and principles enshrined within the term human factors. Such theory underpins a number of rather difficult and somewhat neglected areas of practice such as team working, professionalism and leadership. As clinicians take on more responsibility for shaping the systems within which they work, it becomes essential that they assimilate the experiences of other professionals working in not dissimilar, high-risk disciplines.

This book brings to the table a rich mix of relevant knowledge, anecdote, science and applied psychology. It comprises 18 chapters written by specialist psychologists, engineers, doctors and aviation experts, and links their expertise to the practical aspects of service delivery in a safe but efficient manner. There is a fair amount of science, which I quite liked and will be of practical use; for example, the data on fatigue and performance is clearly relevant to shift designs in clinical medicine. The concept of adaptive vs adapted systems provides much food for thought, particularly when it comes to training and simulation.

Some chapters are easier to read than others and I suspect that many of us will find some of the psychological and management constructs hard going. But overall this is a well-produced and edited book, which will prove a good investment of the time needed to digest it properly. It should be obligatory reading for all medical leaders and managers.

# Sub article: Handbook of Surgical Consent

**Authors:** Rajesh Nair, David J Holroyd

**Table:** 

EXTENT P/H	768 pages, Paperback
PRICE/ISBN	£34.99 9780199595587
PUBLISHER	Oxford University Press (Oxford), 2011
REVIEWER	Sian Pugh
STAR RATING	****

Few doctors have survived their junior years without a copy of the much beloved Oxford Handbook of Clinical Medicine or ‘cheese and onion’ as it is colloquially known. This latest addition to the Oxford Medical Handbook series is aimed at surgical trainees to offer practical guidance on the principles of consent alongside procedure-specific information of the risks and benefits.

The book is divided into specialty-specific chapters that allow the reader to find their subject of interest readily. The concise manner in which it is written permits coverage of a broad range of procedures with well replicated diagrams adding clarity to the text. Complications are comprehensively listed. However, some readers may find the lack of guidance on incidence of these for the majority of procedures unhelpful.

This book certainly attempts to tackle a difficult and challenging topic. The concept that a book can outline what should be included on a surgical consent form for an individual patient may seem impossible to many surgeons. It must be remembered that consent is heavily dependent on the individual patient and so this book should be used as a basis rather than an exhaustive text. Provided it is used with this degree of caution it should be welcomed by trainees as an adjunct to surgical training.
